# Honokiol Protects against Anti-*β*1-Adrenergic Receptor Autoantibody-Induced Myocardial Dysfunction via Activation of Autophagy

**DOI:** 10.1155/2018/1640804

**Published:** 2018-07-18

**Authors:** Xi-qing Wei, Hong-sheng Zhang, Guang-he Wei, Jin-guo Zhang, Yan-yan Du, Hong-yong Tan, Jun Yang

**Affiliations:** ^1^Department of Cardiology, Affiliated Hospital of Jining Medical University, Jining, Shandong 272129, China; ^2^Department of Cardiology, Yantai Yuhuangding Hospital, Affiliated Hospital of Qingdao University, Yantai, Shandong 264000, China; ^3^Qingdao University, Qingdao, Shandong 266071, China

## Abstract

Myocardial diseases are prevalent syndromes with high mortality rate. The exploration of effective interference is important. Anti-*β*1-adrenergic receptor autoantibody (*β*1-AAB) is highly correlated with myocardial dysfunction. The actions and underlying mechanisms of honokiol (HNK) in *β*1-AAB-positive patients await to be unraveled. In this study, we established a rat model of *β*1-AAB positive with myocardial dysfunction. Cardiac function following *β*1-AR-ECII administration was analyzed using the VisualSonics Vevo 770 High-Resolution In Vivo Imaging System. The levels of autophagy-related proteins were detected by Western blotting. Our data revealed that HNK reversed *β*1-AAB-induced effects and protected myocardial tissues from dysfunction. After HNK treatment, the cardiac contractile ability increased and the LDH activity decreased. HNK attenuated myocardial degeneration. In addition, HNK promoted the activation of the AMP-dependent protein kinase/Unc-51-like autophagy activating kinase (AMPK/ULK) pathway and activated autophagy. These results suggest that HNK protects against *β*1-AAB-induced myocardial dysfunction via activation of autophagy and it may be a potentially therapeutic compound for *β*1-AAB-positive myocardial diseases.

## 1. Introduction

Myocardial dysfunction is a complicated syndrome characterized by reduced ejection fraction, biventricular dilatation, and decreased response to fluid administration [1]. Multiple factors, such as sepsis and ischemia/reperfusion, can induce myocardial dysfunction. Cardiovascular abnormalities significantly contribute to the mortality of septic patients [2]. In China, the prevalence of cardiovascular diseases shows an upward trend [3]. Despite the progress in cardiac disease treatment, the mortality rate is still high [4]. Among these mortalities, myocardial dysfunction accounts for approximately 40% [5]. Further exploration of effective intervention for myocardial dysfunction is the key to improving the survival and recovery of patients with cardiac dysfunction.

It has been shown that anti-*β*1-adrenergic receptor autoantibodies (*β*1-AAB) are significantly distributed in the sera of patients with dilated cardiomyopathy and ischemic cardiomyopathy [6, 7]. Binding of specific autoantibodies can lead to increased pulsatility and atrial contractility and activation of the Ca^2+^ channels [8]. Previous studies have demonstrated that the long-term existence of *β*1-AAB causes cardiac function impairments [6, 9]. Cardiomyocyte death is one of the major factors causing heart dysfunction [10]. *β*1-AAB induces the death of adult rat ventricular myocytes in vitro, which may be related to cardiomyocyte apoptosis [11]. It has been shown that *β*1-AAB and its agonist isoproterenol decrease autophagy and are critical for the maintenance of cardiomyocyte homeostasis [12]. Thus, *β*1-AAB can be used to establish a rat model of myocardial dysfunction.

Honokiol (HNK) is a biphenolic compound with antioxidative, anti-inflammatory, and neuroprotective properties [13]. Oral treatment of HNK ameliorates age-related learning and memory dysfunction [14]. In rats, HNK protects the cortex and heart from ischemia/reperfusion injury via inhibition of reactive oxygen species production [15]. HNK induces apoptosis of various tumor cells and reverses epithelial-mesenchymal transition induced by TGF*β* and TNF*α* [13]. Early studies have demonstrated that in rats with myocardial infarction, HNK administration limits infarct size and shows antiarrhythmic effects [16]. These findings suggest that HNK has cytoprotective capabilities. Although HNK has been used to treat various vascular diseases, such as ischemia and stroke [17], whether HNK is beneficial in *β*1-AAB-positive heart diseases is unclear and the underlying mechanisms remain to be explored.

The aim of this study is to investigate the effects of HNK on myocardial injury in a rat model of *β*1-AAB-positive in vivo and to reveal its mechanisms.

## 2. Materials and Methods

### 2.1. Establishment of a Rat Model of *β*1-AAB-Positive and HNK Treatment

Healthy adult male Wistar rats (8 weeks) were obtained from the animal center. Rats were randomly divided into four groups: control group, *β*1-AAB group, *β*1-AAB + HNK group, and *β*1-AAB + vehicle group. *β*1-AR-ECII [a synthetic peptide with 100% rat homology (197~223, H-W-W-R-A-E-S-D-E-A-R-R-C-Y-N-D-P-K-C-C-D-F-V-T-N-R-A, synthesized by GL Biochemical Co., LTD, Shanghai)] was dissolved using Na_2_CO_3_ solution (1 mg/ml) and emulsified using complete Freund's adjuvant (CFA) (1 : 1, Sigma-Aldrich, F5881) [18]. Rats in the *β*1-AAB group were subcutaneously injected with this emulsified antigen (0.4 *μ*g/g). We first injected multiple sites posteriorly along the back of rats. After that, a booster immunization (0.4 *μ*g/g) was given every two weeks. Rats in the control group were injected with normal saline in CFA in the same manner. Each rat blood was collected one day before booster injection, and the formation of *β*1-AAB was detected. Rat sera were extracted using MAbTrap Kit (GE Healthcare, 17–1128-01, Uppsala, Sweden) for the purification of IgG and then analyzed by an ELISA kit. Honokiol was purchased from Sigma-Aldrich (St. Louis, MO) and prepared as described previously [19].

### 2.2. Echocardiography Examinations

We examined cardiac function at 12 h after *β*1-AR-ECII administration by using the VisualSonics Vevo 770 High-Resolution In Vivo Imaging System (VisualSonics Inc., Toronto, Ontario, Canada). The Doppler and M-mode echocardiography was performed according to previous studies [20]. Rats were anesthetized by a mixed gas of oxygen and 2% isoflurane (Rhodia UK Ltd., Avonmouth, Bristol, UK). We performed echocardiography with a 30 MHz center frequency RMV 707 scan head (VisualSonics Inc.). All the echocardiography examinations were performed by the same technician. All the results were from three cardiac cycles.

### 2.3. Measurements of Cardiac Function

As previously described [21], a cannula was inserted into the left ventricle via the right carotid artery. BL-410 recording and analysis system was applied to record and analyze the following parameters: the left ventricular systolic pressure (LVSP), left ventricular end diastolic pressure (LVEDP), and maximal positive and negative values of the instantaneous first derivative of left ventricular pressure (+dP/dt_max_ and −dP/dt_max_).

### 2.4. Measurement of Lactate Dehydrogenase (LDH)

Two hundred thousand cells were plated at the 6-well plates overnight. The culture medium was collected for LDH assays. LDH was measured using a LDH assay kit (Abcam, ab65393). The data were normalized according to the number of cells.

### 2.5. HE Staining

Cardiac tissues were rinsed with PBS and then fixed by 4% paraformaldehyde. The tissues were dehydrated with 30% sucrose in PBS overnight at 4°C and then embedded using embedding medium. Sections were obtained using a freezing microtome. Slides were put into xylene and 100% ethanol in order (each three times, 3 min/time) and then into 95% ethanol (one time, 3 min), 80% ethanol (one time, 3 min) and deionized water (one time, 5 min) in turn. After that, the sections were immersed in hematoxylin (one time, 3 min), rinsed with deionized water and tap water in order (each one time, 5 min). Acid ethanol was used to destain the slides. After rinsing with tap water (two times, 1 min) and deionized water (one time, 2 min), sections were immersed in eosin (one time, 30 seconds). After rinsing with 95% and 100% ethanol (each three times, 5 min/time), slides were put into xylene (three times, 15 min/time). At last, slides were mounted with permount.

### 2.6. Immunofluorescence

Cultured H9c2 cells were exposed to 40 *μ*M honokiol for 72 h and were washed twice using PBS. After that, cells were fixed by 4% paraformaldehyde for 15 min and then washed using PBS. Next, cells were incubated using 0.2% Triton in PBS for 0.5 hours. After that, cells were blocked by blocking buffer for 1 h at room temperature. Last, cells were incubated with LC3B antibody (1 : 400; Cell Signaling, 2775) in a humidified box overnight at 4°C. On the second day, cells were first washed using PBS and then incubated with a second antibody for 2 h. After washing with PBS, we added 2-(4-amidino-phenyl)-6-indolecarba-midine dihydrochloride (DAPI, Beyotime Biotech, C1005) solution to stain the nuclei for 3 min. Cells were washed using PBS and then sealed with a coverslip. The images were captured using laser confocal microscopy (OLYMPUS, FV1000, USA).

### 2.7. Antibodies and Reagents

LC3 and p62 antibodies were purchased from Novus Biologicals (Abcam, ab48394) and Enzo Life Sciences (Abcam, ab56416), respectively. Other antibodies were obtained from Cell Signaling (Boston, MA, USA).

### 2.8. Western Blot

Extracts from tissues and cultured cells were prepared as previously described [22]. The homogenates were centrifuged (14,000 rpm, 30 min) at 4°C, and the supernatants were collected. We determined protein concentrations using the BCA Protein Assay (Thermo Scientific, Rockford, IL). Protein lysates (approximately 40 *μ*g) were separately loaded on 10% polyacrylamide gels and then transferred onto polyvinylidene difluoride membranes. The membranes were blocked by blocking buffer (5% milk in PBS) for 30 min at room temperature. After that, the primary antibodies (1 : 10,000) were added and incubated overnight at 4°C. The membranes were washed in PBST (PBS with 0.2% Tween-20) and incubated with secondary antibodies (1 : 10,000) for 1 h at room temperature. The membranes were washed in PBST, and the images were captured using the Odyssey Infrared Imaging System (Li-COR Biotechnology).

### 2.9. Statistical Analysis

Data were shown as the mean ± standard deviation (SD). We performed the statistical analysis by SPSS software (version 15.0, SPSS Inc., Chicago, IL, USA). We used Student's *t*-test to compare the means of two independent groups and applied one-way ANOVA with a Bonferroni post hoc test for more than two independent samples. Statistical significance was defined when *P* < 0.05.

## 3. Results

### 3.1. A Rat Model of Long-Term Maintenance of *β*1-AAB Was Established

**Figure 1:**
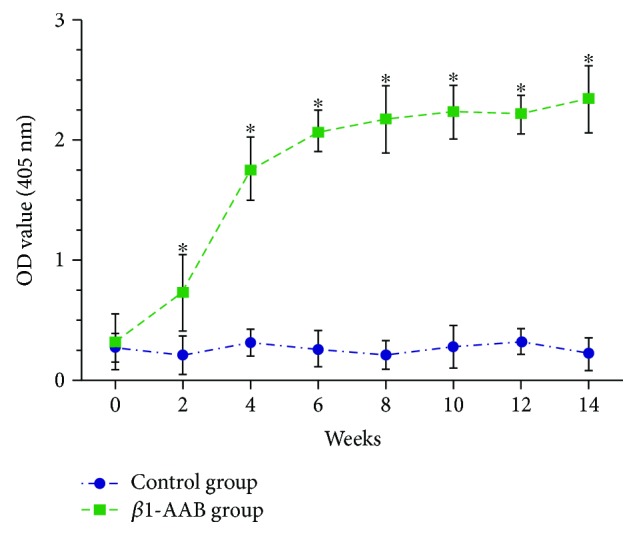
Immunization against *β*1-AR-ECII induced a high-level accumulation of *β*1-AAB in the blood. *β*1-AR-ECII (*β*1-AAB group) or saline (control group) were subcutaneously injected into adult rats. Antibody titer in the blood was defined by OD value. At the beginning, *β*1-AABs in the two groups were similarly low. However, β1-AABs were sharply increased in the first four weeks and maintained high in the *β*1-AR-ECII treatment group. Data were expressed as mean ± SD (*n* = 12 per group). ^∗^*P* < 0.05.

To establish a rat model of long-term maintenance of *β*1-AAB, *β*1-AR-ECII or saline were subcutaneously injected into adult rats. *β*1-AAB titer in the blood was then defined by OD value. At the beginning of immunization, the protein levels of *β*1-AABs in the sera were both very low in the two groups (Figure 1). Compared to the saline treatment group, *β*1-AABs were sharply increased in the first four weeks and maintain a high level later in the *β*1-AR-ECII treatment group (Figure 1). These data indicate that we have successfully established a rat model of long-term high-level maintenance of *β*1-AAB.

### 3.2. HNK Prevented *β*1-AAB-Provoked Myocardial Dysfunction

**Figure 2:**
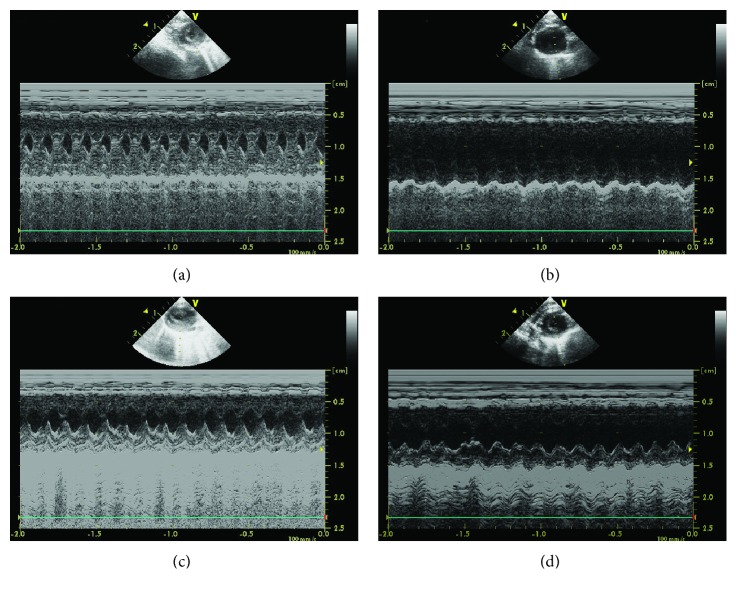
Honokiol ameliorated cardiac contractility suppressed by *β*1-AAB. Representative M-mode echocardiograms were obtained from the rats at 12 h after each treatment. (a) Saline treatment (control group); (b) *β*1-AR-ECII administration (*β*1-AAB group); (c) *β*1-AR-ECII plus HNK administration (*β*1-AAB + HNK group); and (d) *β*1-AR-ECII plus saline administration (*β*1-AAB + vehicle group). HNK: honokiol.

To investigate the cardiac ventricular function, echocardiography was used to examine it at 12 h following injection with saline or *β*1-AR-ECII [23]. As shown in Figure 2, the M-mode echocardiography evaluation showed that compared to the control group, significant suppression of cardiac ventricular contractility was observed in the group with *β*1-AR-ECII treatment (Figures 2(a) and 2(b)). In contrast, treatment with HNK obviously reversed the decrease of myocardial contraction, whereas there was no significant difference between the *β*1-AR-ECII treatment and *β*1-AR-ECII plus saline treatment groups (Figures 2(c) and 2(d)).

**Figure 3:**
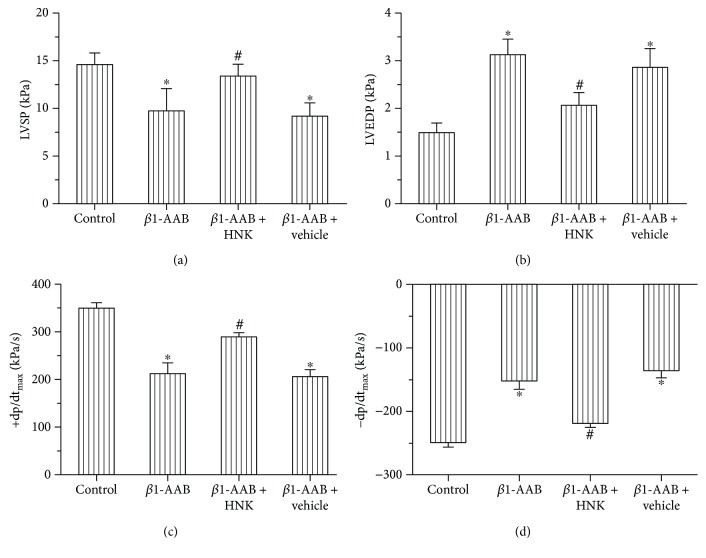
Honokiol protected cardiac systolic and diastolic function. Rats were randomly divided into the control group (saline treatment), *β*1-AAB group (*β*1-AR-ECII treatment), *β*1-AAB + HNK group (*β*1-AR-ECII plus HNK treatment), and *β*1-AAB + vehicle group (*β*1-AR-ECII plus saline treatment). LVSP (a), LVEDP (b), +dP/dt_max_ (c), and −dP/dt_max_ (d) were measured. In the *β*1-AAB group, LVSP and +dP/dt_max_ decreased and LVEDP and −dP/dt_max_ increased. These values changed in the opposite direction in the *β*1-AAB + HNK group, whereas there was no difference in the *β*1-AAB + vehicle group. Data were expressed as mean ± SD (*n* = 12 per group). ^∗^*P* < 0.05 relative to the control group. ^#^*P* < 0.05 relative to the *β*1-AAB group. HNK: honokiol.

To examine the cardiac systolic and diastolic function, we measured the parameters below 12 weeks after active immunization: left ventricular systolic pressure (LVSP), left ventricular end diastolic pressure (LVEDP), and maximal positive and negative values of the instantaneous first derivative of left ventricular pressure (+dP/dt_max_ and −dP/dt_max_). LVSP and +dP/dt_max_ represent systolic function. LVEDP and −dP/dt_max_ indicate diastolic function. Compared to the control group, the values of LVSP and +dP/dt_max_ significantly reduced and the values of LVEDP and −dP/dt_max_ obviously increased in the *β*1-AAB-positive group (Figure 3). HNK addition significantly reversed all these effects induced by β1-AAB, but there was no difference in the saline cotreatment group (Figure 3). These data indicate that the continuous immunization significantly inhibits cardiac ventricular contractility, but HNK-combined therapy obviously ameliorates the suppression of ventricular contractility and reverses cardiac dysfunction.

### 3.3. HNK Reduced Lactate Dehydrogenase (LDH) Activity and Attenuated Myocardial Degeneration

**Figure 4:**
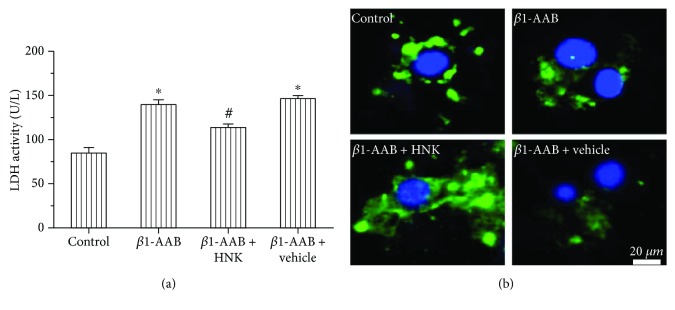
Honokiol reduced LDH activity and increased LC3 staining. H9c2 cells were randomly divided into the control group (saline treatment), *β*1-AAB group (*β*1-AR-ECII treatment), *β*1-AAB + HNK group (*β*1-AR-ECII plus HNK treatment), and *β*1-AAB + vehicle group (*β*1-AR-ECII plus saline treatment). (a) The serum level of LDH in each group was determined. It increased in the *β*1-AAB group and significantly reduced in the *β*1-AAB + HNK group. (b) Representative immunofluorescent images of LC3 (green) and DAPI (blue) in each group. The staining intensity of LC3 reduced in the β1-AAB group and increased in the *β*1-AAB + HNK group. Data were shown as mean ± SD (*n* = 6 per group). ^∗^*P* < 0.05 relative to the control group. ^#^*P* < 0.05 relative to the *β*1-AAB group. HNK: honokiol; LDH: lactate dehydrogenase.

**Figure 5:**
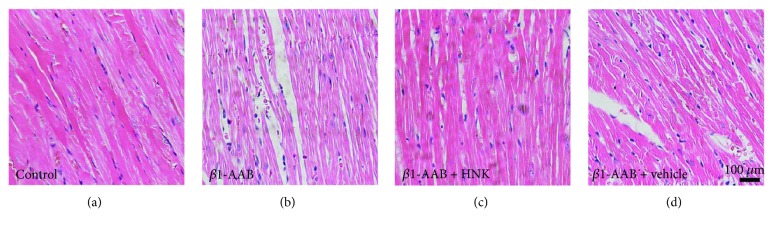
Honokiol attenuated the degeneration of cardiac tissue. (a), (b), (c), and (d) represented the HE staining images of cardiac tissue sections from the control, *β*1-AAB, *β*1-AAB + HNK, and *β*1-AAB + vehicle groups, respectively. (b) There was extensive myocardial degeneration in the *β*1-AAB group. (c) A significant improvement of myocardial pathological features was observed in the *β*1-AAB + HNK group. (d) There was no obvious amelioration of the pathological impairments in the *β*1-AAB + vehicle group.

To detect the degree of cell injury, we measured the activity of LDH in each group. There was a significant elevation of LDH in the *β*1-AAB group, and HNK addition remarkably decreased the level of LDH (Figure 4(a)). There was no significant difference between the *β*1-AAB and *β*1-AAB + vehicle groups (Figure 4(a)). The results of HE staining in the *β*1-AAB group two weeks after active immunization revealed that the myocardial cells underwent extensive pathological changes, including hypertrophy, disorganization, and more vacuoles with varying shapes (Figure 5(b)). These pathological changes were obviously ameliorated in the *β*1-AAB + HNK group, but there was no significant change in the *β*1-AAB + vehicle group (Figures 5(c) and 5(d)). These results indicate that HNK protects myocardial cells from breakage and degeneration directly or indirectly induced by high-level *β*1-AAB.

### 3.4. HNK Activated Autophagy Inhibited by *β*1-AAB

**Figure 6:**
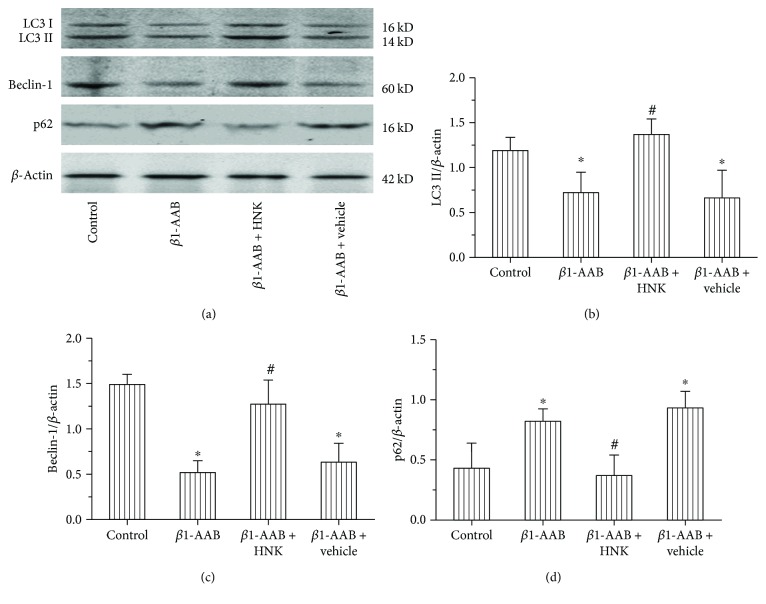
Honokiol activated autophagy in myocardial tissues after passive immunization. (a) Representative Western blot images of autophagic markers LC3I, LC3II, Beclin-1, and p62 in cardiac tissue lysates from each group. (b–d) Quantification and comparison of Western blot band values from (a). In the *β*1-AAB group, LC3II and Beclin-1 decreased and p62 increased. These protein levels changed in the opposite direction in the *β*1-AAB + HNK group. Data were presented as mean ± SD (*n* = 12 per group). ^∗^*P* < 0.05 relative to the control group. ^#^*P* < 0.05 relative to the *β*1-AAB group. HNK: honokiol.

Microtubule-associated protein 1 light chain 3 I (LC3I) and LC3II are markers of autophagy. We demonstrated that the expression of LC3 in the *β*1-AAB group was lower than that in the control group by immunofluorescence staining (Figure 4(b)). However, the staining intensity was stronger in the *β*1-AAB + HNK group and it remained low in the *β*1-AAB + vehicle group (Figure 4(b)). Notably, the Western blot data obtained after active immunization for four weeks were consistent with these results (Figures 6(a) and 6(b)). Beclin-1 is an important gene for autophagy. As p62 was degraded within the autolysosomes, it was usually used as an indicator of autophagic flux. To find out the effect of HNK on autophagy, the protein levels of LC3I and LC3II, Beclin-1, and p62 in the heart tissue of each group were detected. The protein levels of LC3I and LC3II and Beclin-1 in the *β*1-AAB group significantly reduced, while the level of p62 protein obviously increased (Figures 6(a)–6(d)). Compared to those of the *β*1-AAB group, the protein levels of the *β*1-AAB + HNK group had the opposite change, while the addition of normal saline had no obvious effect on the protein level (Figure 6(a)-6(d)). These data suggest that HNK is beneficial to the heart tissue by activating autophagy in a rat model of long-term maintenance of *β*1-AAB.

### 3.5. HNK Promoted the Activation of the AMP-Dependent Protein Kinase/Unc-51 Like Autophagy Activating Kinase Pathway

**Figure 7:**
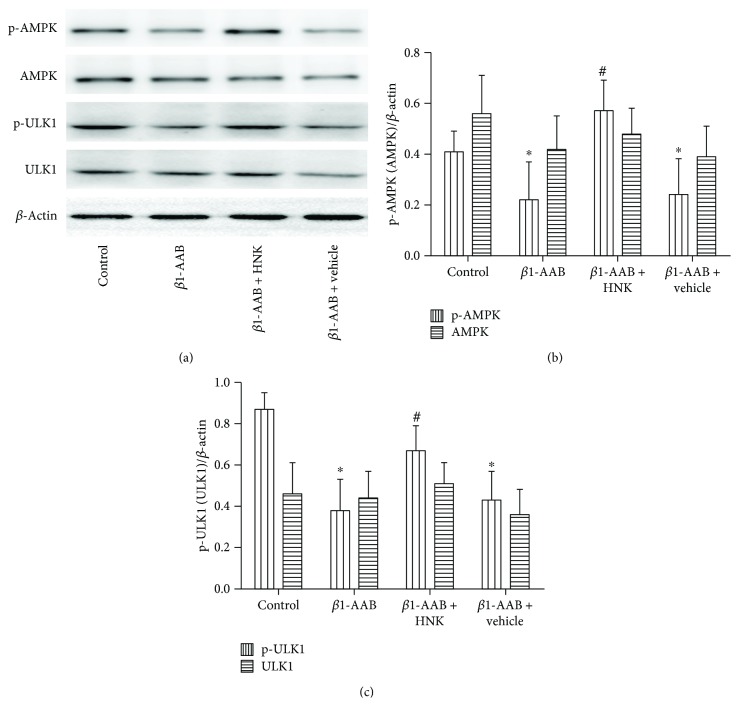
Honokiol activated the AMPK/ULK1 pathway. (a) Representative Western blot images of p-AMPK (Thr172), AMPK, p-ULK1 (Ser555), and ULK1 in cardiac tissue lysates from each group. (b) Quantification and comparison of p-AMPK and AMPK band values from (a). (c) Quantification and comparison of p-ULK1 and ULK1 band values from (a). The phosphorylation of AMPK and ULK1 were inhibited by *β*1-AAB and activated by HNK. Data were presented as mean ± SD (*n* = 12 per group). ^∗^*P* < 0.05 relative to the control group. ^#^*P* < 0.05 relative to the *β*1-AAB group. HNK: honokiol.

AMPK (AMP-dependent protein kinase) is a major energy-sensitive kinase regulating many cell processes, including macroautophagy. The phosphorylation of Unc-51-like autophagy activating kinase (ULK) involves the formation of autophagosome [24]. To investigate whether the AMPK/ULK pathway was involved in the regulation of HNK, we used Western blot to measure the protein levels and phosphorylation patterns of AMPK and ULK four weeks after active immunization. We found that the levels of AMPK and ULK were not changed among the groups (Figures 7(a)–7(c)). However, the levels of phosphorylated AMPK and phosphorylated ULK were remarkably reduced in the *β*1-AAB group, compared to the control group (Figures 7(a)–7(c)). HNK cotreatment significantly increased these phosphorylated protein levels, but there was no obvious difference in the *β*1-AAB + vehicle group (Figures 7(a)–7(c)). These results indicate that the AMPK/ULK pathway activated by HNK may be the intrinsic mechanism of increased autophagy.

## 4. Discussion

This study is to explore the effects of HNK on *β*1-AAB-positive heart diseases and the potential mechanisms. We established a rat model of long-term maintenance of *β*1-AAB and demonstrated that the combination therapy of HNK prevented myocardial dysfunction and decreased LDH activity. We revealed that HNK was capable of attenuating myocardial degeneration. Additionally, we showed that HNK cotreatment promoted the activation of the AMPK/ULK pathway and activated autophagy inhibited by high-level of *β*1-AAB. To the best of our knowledge, this is the first time to demonstrate that HNK protects against *β*1-AAB-induced myocardial dysfunction. Furthermore, we have revealed that autophagy and the AMPK/ULK signaling pathway may underlie the benefits.

*β*1-AAB was often detected in patients with various heart diseases [25]. It has been shown that *β*1-AAB aggravates heart function [9]. HNK exerts protective functions in many vascular diseases [26]. Here, a rat model of long-term high-level maintenance of *β*1-AAB was established (Figure 1). The continuous immunization caused significant suppression of cardiac ventricular contractility, and HNK cotreatment obviously ameliorated the adverse effect (Figures 2 and 3). These data indicate that HNK directly or indirectly regulates myocardial cell contractility. Cellular energy is critical for cell contractility [27]. The physiological maintenance of the respiratory chain needs the integrity of mitochondrial membrane [28]. *β*1-AAB leads to the disruption of the electrochemical gradient ΔΨm that reflects the integrity of the inner mitochondrial membrane [18]. HNK protects myocardial mitochondria from lipid peroxidation and decreases the myocardial infarct size caused by coronary occlusion [26]. In mice, HNK prevents cardiac hypertrophy by activating mitochondrial Sirt3 [29]. In our model, we propose that HNK may protect myocardial mitochondria from *β*1-AAB-induced impairments. It is reported that LDH leaks from damaged cells [30]. We demonstrated that HNK cotreatment inhibited the increase of LDH activity in the *β*1-AAB-positive group (Figure 4(a)). Myocardial degeneration was also attenuated in the HNK cotreatment group (Figure 5). These results indicate that HNK may protect myocardial cells via mitochondrial regulation. A recent publication demonstrates that HNK protects the heart from Dox cardiotoxicity via improving mitochondrial function by not only repressing mitochondrial protein acetylation but also enhancing PPAR*γ* activity in the heart [31].

Autophagy is a process of degradation of abnormal cellular components and necessary for maintaining cellular homeostasis [32]. Autophagy can be induced by mitochondrial impairments and inhibited by a *β*-adrenergic agonist [33]. LC3 is a component of the vacuoles during autophagy [34]. LC3I is a cytoplasmic, and LC3II is localized in the membrane of autophagosome. The increase of LC3II reflects the accumulation of autophagosomes, indicating the induction of autophagy [35]. Beclin-1 plays an important role in mediating the localization of autophagic proteins to preautophagosome [36]. P62 is degraded within the autolysosomes, the increase of which reflects the inhibition of autophagy [37]. Thus, LC3, Beclin-1, and p62 can be used as good indicators for autophagy analysis. The immunostaining and Western blot data showed that the levels of LC3II and Beclin-1 decreased and the level of p62 increased in the *β*1-AAB-positive group. The addition of HNK significantly counteracted the effects (Figures 4(b) and 6). It is worth noting that HNK treatment promotes the recovery of systolic function and the inhibition of diastolic function (Figure 3). These data suggest that HNK protects myocardial functions by activating autophagy. Consistent with this, previous studies have shown that reduced autophagy contributes to heart disorders [38] and upregulated autophagy is remarkably beneficial for impaired cardiac function [39].

A complex signaling network regulates autophagy, most of which correlates with the AMP-activated protein kinase (AMPK)/ULK pathway [40]. It has been reported that Beclin-1 activity depends on the phosphorylation of AMPK [41]. Phosphorylated ULK1 is negatively regulated by mTOR that can be inhibited by AMPK [41]. HNK increased phosphorylated forms of both AMPK and ULK (Figure 7), indicating the underlying mechanism of increased autophagy. AMPK activation is mainly regulated by the phosphatases and kinases, and its distribution is tissue-specific. For instance, LKB1 kinase exists in the peripheral tissues, while CaMKK2 kinase is expressed in neurons [42]. Thus, it is likely that HNK activates AMPK via LKB1.

Combined with these data, we propose that HNK benefits cardiac tissue not by directly reducing the level of *β*1-AAB in the sera.

## 5. Conclusion

In conclusion, this present study showed that HNK protected hearts from dysfunction in a rat model of *β*1-AAB-positive, possibly by reducing the activity of LDH, activating the AMPK/ULK pathway, and promoting autophagy. These results suggest that HNK may be a potentially therapeutic compound to cure *β*1-AAB-positive heart diseases. These preliminary observations provide novel insights into the prevention of *β*1-AAB-positive cadiovascular diseases. The detailed mechanisms for these additive effects remain unclear. It is worthwhile to investigate the effect of HNK on the mitochondria or energy metabolism in the future.

## Data Availability

All data generated or analyzed in this study are included in this published article.
